# Vitamin D add on the standard treatment for myasthenia gravis symptoms following total gastrectomy: a case report

**DOI:** 10.1186/s12883-024-03687-z

**Published:** 2024-06-05

**Authors:** Tao Zhang, Junhong Zhong, Xu Ji, Jingqing Sun, Yingxue Cui, Shaosong Wang

**Affiliations:** 1 Department of Acupuncture, Beijing Hospital of Traditional Chinese Medicine, Capital Medical University, No.23 Museum Back Street, Dongcheng District, Beijing, 100010 China; 2 Department of Neurology, Inner Mongolia Hospital affiliated to Beijing Hospital of Traditional Chinese Medicine, Bayannur city, Inner Mongolia Autonomous Region, 015000 China

**Keywords:** Case report, Myasthenia gravis, Vitamin D, Gastrectomy

## Abstract

**Background:**

Myasthenia gravis (MG) is a long-term autoimmune disorder that affects the neuromuscular junction, causing muscle weakness and fatigue as its primary clinical features. Vitamin D is crucial for both the autoimmune response and skeletal muscle function.

**Case presentation:**

Here, we presented a case report documenting the substantial improvement in symptoms experienced by a patient who underwent subtotal gastrectomy for gastric cancer following high-dose Vitamin D supplementation. The patient developed generalized MG two months after the surgery and did not respond adequately to pyridostigmine therapy, experiencing a progressive deterioration of the condition. A significant reduction in vitamin D concentration was observed following subtotal gastrectomy. In response, high-dose vitamin D supplementation was administered to the patient. Within one week of treatment, swallowing symptoms improved, enabling the consumption of a small amount of liquid food. By the second week, substantial swallowing and neck function improvements were evident. After one month, the patient regained the ability to straighten the neck while walking and consumed a regular diet despite persistent difficulties chewing hard food.

**Conclusions:**

This case underscores the therapeutic potential of vitamin D in alleviating MG symptoms, particularly in individuals with compromised vitamin D levels following gastrectomy. The observed improvements present a new perspective on the possible involvement of vitamin D supplementation in the management of postoperative MG cases.

## Background

Myasthenia gravis (MG) is an autoimmunological condition impacting neuromuscular junction postsynaptic membrane acetylcholine signaling, mediated by antibodies including acetylcholine receptor (AchR) antibody, cellular immunity, and complement. It presents as acquired skeletal muscle weakness [1]. The participation of cytokines and activated T and B cells is crucial in producing pathogenic autoantibodies and the onset of inflammation at the neuromuscular junction in MG [2]. The activation of the immune response by dysfunctional regulatory T cells (Tregs)has been shown to contribute to the exacerbation of MG pathogenesis potentially, highlighting the significant involvement of T cells in the disease’s development and progression [3]. Research indicates that the defect in immune regulation in MG is predominantly localized in isolated Tregs [4, 5].

The primary metabolites of vitamin D, 25-hydroxyvitamin D [25(OH)D] and 1,25-dihydroxyvitamin D [1,25(OH)2D], exhibit potent immunomodulatory properties. Vitamin D’s immunomodulatory effects include direct inhibition of effector T cells and induction of Tregs to suppress the production of inflammatory cytokines, indicating its potential significance in T-cell regulation [6]. Vitamin D deficiency is associated with various chronic autoimmune diseases such as multiple sclerosis (MS) [7], systemic lupus erythematosus (SLE) [8], and rheumatoid arthritis (RA) [9]. Recent studies and systematic reviews have demonstrated significantly lower plasma 25(OH)D levels in patients with MG relative to healthy controls, suggesting the importance of monitoring 25(OH)D levels in MG patients [10].

Limited evidence currently links vitamin D deficiency with the initiation or deterioration of MG. However, we present a case of a patient who developed gastric cancer and subsequently underwent total gastrectomy. Two months after the surgery, the patient experienced facial and systemic muscle weakness, leading to a diagnosis of MG based on symptoms, signs, immunologic tests, and electrophysiological examination. Despite initial treatment with pyridostigmine bromide, the patient’s clinical response was inadequate, and symptoms deteriorated progressively. Prior research has established a link between total gastrectomy and vitamin D deficiency [11, 12] and an connection between MG symptoms and vitamin D levels [13]. Given the potential impact of total gastrectomy on vitamin D absorption, the patient’s serum 25-hydroxyvitamin D level was found to be notably reduced. Subsequent administration of high-dose vitamin D resulted in a marked improvement in symptoms and enhanced muscular functions. This case report offers valuable insights into how vitamin D supplementation may potentially manage postoperative MG cases.

## Case report

In April 2021, a 62-year-old male patient was diagnosed with gastric cancer and subsequently underwent multiple chemotherapy treatments at Peking Union Medical College Hospital. On January 10, 2022, he underwent a total gastrectomy at the same hospital. In early March 2022, the patient began experiencing blepharoptosis, followed by progressive dysphagia, neck weakness, and fatigue, exacerbated after physical exertion. Symptoms were milder in the morning and worsened in the evening, prompting the patient to seek medical attention at Peking Union Medical College Hospital, where he underwent relevant examinations. At admission, a paraneoplastic screen was performed and found negative for paraneoplastic syndrome. The head and neck magnetic resonance imaging (MRI) and computerized tomography angiography (CTA) revealed no abnormalities in intracranial lesions or vascular malformations. Thymus computerized tomography (CT) also showed no abnormalities. Electromyographic examination indicated stimulation of the bilateral facial nerve and accessory nerve by repetitive frequency stimulation, and the neostigmine test yielded a positive result, leading to a diagnosis of MG. The patient was prescribed oral pyridostigmine bromide at a dose of 60 mg three times a day, which initially relieved subjective symptoms. However, after two weeks, the patient experienced worsening symptoms, including difficulty straightening the neck and swallowing, faint and low voice, and overall weakness. Following daily activities, the patient’s symptoms deteriorated, necessitating the use of a wheelchair when venturing outside. Despite the addition of 240 mg of pyridostigmine bromide daily, the symptoms were not alleviated.

On April 1st, 2022, the patient was admitted to our department for inpatient care and denied a history of hypertension, other immune system disorders, and exposure to potentially harmful medications. The patient also reported regular outdoor activities to receive sunlight. Due to the potential risk of gastrointestinal bleeding post-total gastrectomy, glucocorticoid treatment was deemed unsuitable, and gamma globulin therapy was declined due to financial constraints.

To investigate a potential causal relationship between vitamin D, total gastrectomy and MG in the patient [11–13], we conducted tests to analyze the spectrum of acetylcholine receptor antibodies and the serum vitamin D level. The patient’s AchR antibody value (measured by enzyme-linked immunosorbent assay, ELISA) was 1.865 nmol/L, significantly exceeding the normal range of < 0.625 nmol/L. The Titin antibody value (ELISA) was 0.504 nmol/L, also significantly higher than the normal range of < 0.472 nmol/L. Additionally, compared to the typical range of > 30 ng/ml, the serum vitamin D (25-OH) level was 6.64 ng/ml, signifying a significant deficiency.

After observing a significant decline in the patient’s vitamin D levels, we commenced high-dose vitamin D supplementation. With the patient weighing 47 kg, we started with a daily dosage of 2400 IU of vitamin D in the first week, subsequently elevating the dosage to 4000 IU daily after seven days. After a month, the supplementation was reduced to 2400 IU daily for maintenance. Simultaneously, the patient continued the regimen of pyridostigmine bromide at a dosage of 60 mg thrice daily.

**Fig. 1:**
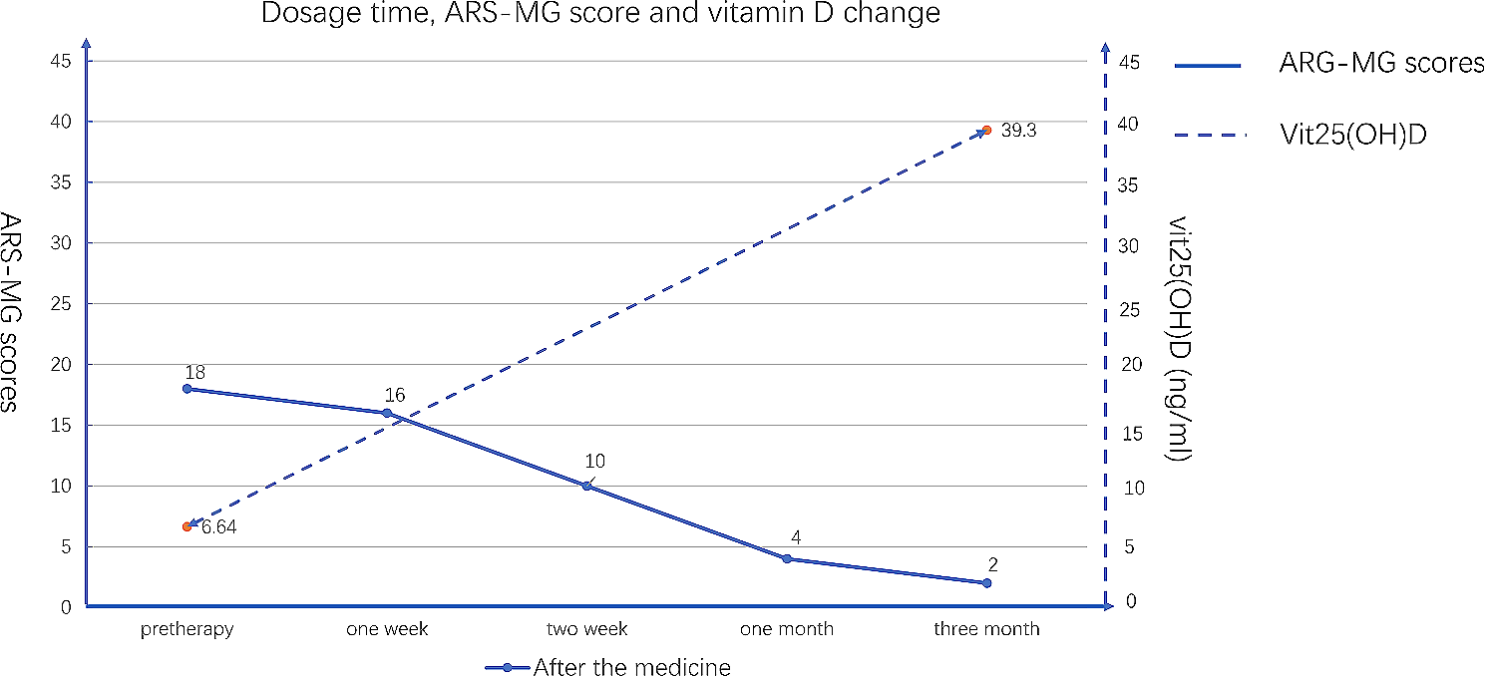
Relationship between the changes in ARS-MG scores and Vit25(OH)D dosage throughout treatment

The severity of symptoms before and after treatment was assessed using the Absolute and Relative Score of MG (ARS-MG) scoring scale [14]. The relationship between the changes in the severity of symptoms and vitamin (25-OH) D concentration is illustrated in Fig. 1. As the treatment went on, the vitamin (25-OH) D concentration got back to normal range, and the symptoms showed great improvements (the ARS-MG score decreased from 18 [before treatment] to 2 [after treatment] ). After one week of treatment, the patient experienced improved swallowing symptoms and was able to consume a small amount of liquid food. By the end of the second week, there was a notable enhancement in swallowing and neck function. After one month of medication, the patient could walk with a straightened neck and consume a regular diet, albeit with ongoing challenges in chewing hard food. By the third month, the patient could independently walk 300–500 m without rest and had improved chewing ability. The strength of the neck-lifting muscle had also recovered, enabling the patient to perform daily activities without difficulty. The dosage of vitamin D remained unchanged, and symptom progression was continuously monitored. A re-evaluation of vitamin D concentration (25-OH) three months later revealed a level of 39.3 ng/mL (normal range: >30 ng/ml).

The MG-activities of daily living profile (MG-ADL) was also used to assess the patient before and after the treatment. Before treatment, the MG-ADL score was 18, and decreased to 15 after one-week treatment. The MG-ADL scores were 8, 3, and 1 respectively at the 2nd week, 4th week, and 12th week after the treatment. The MG-ADL score changes indicated the great improvement of the patient.

## Discussion

MG is generally an autoimmune condition that is caused by autoantibodies targeting the AChR. The role of anti-AChR antibodies in the development of MG has been firmly established, and the production of these autoantibodies relies on T-cell activity. It is crucial to regulate potentially pathogenic T cells, and the Treg is one of the primary regulators of effector T cells [5]. A mounting body of evidence has demonstrated that Tregs are functionally defective in patients with MG, emphasizing their pivotal role in immune regulation. Recent studies in healthy individuals have supported the hypothesized immune-regulating impact of vitamin D, which is believed to be connected to increased regulatory T cells [10, 15].

Vitamin D has potential relationship with MG: first, it modulates autoimmune responses, and second, it maintains muscle function by acting on vitamin D receptors in muscle [16]. Experimental studies have demonstrated that 25(OH)D, the metabolically active form of vitamin D, exerts its immunomodulatory effects by increasing the number of Tregs [17]. Additionally, it is thought that 1,25(OH)2D3 works by reducing proinflammatory interleukin-2 and interferon-γ expression, inhibiting the proliferation and differentiation of T helper cell 1, regulating cytokine production, stimulating T helper cell two by upregulating anti-inflammatory cytokine production, inhibiting the development of Th17 cells, and inhibiting interleukin-17, which induces the proliferation of regulatory T cells [18]. Therefore, studies suggest a link between vitamin D concentration and the onset of MG. Compared with healthy individuals, MG patients are at a higher risk of having insufficient levels of vitamin D, and those with more severe diseases tend to have even lower vitamin D levels [19]. Further research is necessary to determine how vitamin D contributes to the pathogenesis of MG and the potential benefits of vitamin D supplementation.

Some studies have shown that the vitamin D receptor (VDR) acts as a transcription factor and the immunomodulatory impact of 1,25(OH)2D3 is facilitated through its binding to VDR [20]. Notably, the VDR gene Tru9I (rs757343) polymorphism has been closely linked to the susceptibility of MG in females aged over 15 years [21, 22]. Furthermore, in the Chinese Han population, there is a potential association between rs731236 and adult non-thymoma AChRAb-negative MG patients, implying a connection between vitamin D and the onset of MG [23, 24].

The initial clinical study on vitamin D deficiency in individuals with MG was published in 2012 [25]. The findings revealed that MG patients had significantly lower plasma 25(OH)D levels in comparison to healthy controls, attributed to reduced sun exposure and high body mass index (BMI). Administering vitamin D3 supplementation at a dosage of 800 IU/day for 2.5–10 months (mean 6 months) to MG patients without prior vitamin D3 supplementation demonstrated positive effects on autoimmune response and fatigue scores. Subsequent research has suggested a potential effect for vitamin D in MG. However, the available information on the vitamin D status and the recommended supplemental dosage for MG patients is limited and inconclusive. Some studies investigating vitamin D in individuals with MG have reported contradictory findings regarding the prevalence of deficient or insufficient vitamin D levels. Some studies have produced conflicting results and have failed to consider the association between outdoor activities and vitamin D intake [26–29].

The patient was administered a daily vitamin D supplementation, with the dosage ranging from 2400 to 4000 IU. However, high-dose vitamin D therapy for autoimmune diseases does not have sufficient evidence to prove its long-term safety. Although trials are investigating supraphysiological doses of vitamin D for MS, similar studies for other conditions are limited [13]. The 2022 guidelines for MG do not provide specific recommendations for the treatment of vitamin D, and the vitamin D status in MG patients remains uncertain [30, 31]. Existing literature suggests that patients on long-term high-dose vitamin D, usually between 80,000 and 120,000 IU/day, do not experience serious adverse reactions [13]. However, no research currently supports larger doses, and further investigation is necessary.

In this reported case, the patient had a documented history of total gastrectomy, which is frequently linked to reduced vitamin D levels. High-dose vitamin D treatment was administered without interference with concurrent medications, resulting in an evident improvement of the patient’s muscle weakness symptoms. While this article constitutes a single case report and thus cannot furnish conclusive evidence for clinical practice, it suggests that physicians should consider monitoring and supplementing vitamin D levels in patients exhibiting MG symptoms following gastrointestinal surgery. This approach may yield improved clinical outcomes and increased longevity.

## Data Availability

The datasets analysed during the current study are available from the corresponding author on reasonable request.

## Abbreviations

MG: Myasthenia gravis
AchR: Acetylcholine receptor
[25(OH)D]: 25-hydroxyvitamin D
MS: Multiple sclerosis
SLE: Systemic lupus erythematosus
RA: Rheumatoid arthritis
MRI: Magnetic resonance imaging
CTA: Computerized tomography angiography
